# Fecal Colonization With Multidrug-Resistant *E. coli* Among Healthy Infants in Rural Bangladesh

**DOI:** 10.3389/fmicb.2019.00640

**Published:** 2019-04-02

**Authors:** Mohammad Aminul Islam, Mohammed Badrul Amin, Subarna Roy, Muhammad Asaduzzaman, Md. Rayhanul Islam, Tala Navab-Daneshmand, Mia Catharine Mattioli, Molly L. Kile, Karen Levy, Timothy R. Julian

**Affiliations:** ^1^Food Microbiology Laboratory, Laboratory Sciences and Services Division, International Centre for Diarrhoeal Disease Research, Bangladesh (ICDDR,B), Bangladesh, India; ^2^School of Chemical, Biological and Environmental Engineering, Oregon State University, Corvallis, OR, United States; ^3^Division of Foodborne, Waterborne, and Environmental Diseases, Centers for Disease Control and Prevention, Atlanta, GA, United States; ^4^Department of Environmental Health, Rollins School of Public Health, Emory University, Atlanta, GA, United States; ^5^School of Biological and Population Health Sciences, Oregon State University, Corvallis, OR, United States; ^6^Eawag, Swiss Federal Institute of Aquatic Science and Technology, Dübendorf, Switzerland; ^7^Swiss Tropical and Public Health Institute, Basel, Switzerland; ^8^University of Basel, Basel, Switzerland

**Keywords:** colonization, multidrug-resistant, *E. coli*, ESBL, third generation cephalosporins

## Abstract

Third generation cephalosporins (3GC) are one of the main choices for treatment of infections caused by multidrug-resistant (MDR) Gram-negative bacteria. Due to their overuse, an increasing trend of resistance to 3GC has been observed in developing countries. Here, we describe fecal colonization of 3GC-resistant (3GCr) *Escherichia coli* in healthy infants (1–12 months old) living in rural areas of Bangladesh. We found that stool samples of 82% of infants (*n* = 100) were positive for 3GCr *E. coli* with a mean ± standard deviation of 6.21 ± 1.32 log_10_ CFU/g wet weight of stool. 3GCr *E. coli* encompasses an average one third (33%) of the total *E. coli* of stool. Almost 77% (*n* = 63) of these 3GCr *E. coli* were MDR (or resistant to ≥3 classes of antibiotics). Around 90% (*n* = 74) of 3GCr *E. coli* were extended spectrum beta-lactamase (ESBL)-producing in which *bla*_CTX–M–group–1_ was the predominant (96%, *n* = 71) ESBL-gene followed by *bla*_TEM_ (41%, *n* = 30) and *bla*_OXA–1_ (11%, *n* = 8). A significant proportion (26.5%, *n* = 22) of 3GCr *E. coli* was pathogenic, comprising two types, enteroaggregative (EAEC, *n* = 19) and enteropathogenic (EPEC, *n* = 3). Colonization of 3GCr *E. coli* in infant guts was not associated with demographic characteristics such as age, sex, mode of delivery, maternal and infant antibiotic use, disease morbidity, and feeding practices. The high rate of colonization of 3GCr *E. coli* in infants’ guts is a serious public health concern which needs immediate attention and warrants further studies to explore the cause.

## Introduction

The rapid rise of multidrug-resistant (MDR) bacterial infections is a major public health concern and a growing threat to the global health security. Unregulated use of broad spectrum antibiotics and widespread reservoirs of these pathogens are main contributors to this problem (Hilty et al., 2012). Broad spectrum antibiotics, in particular third generation cephalosporins (3GC), are among the most frequently prescribed drugs for the treatment of infections caused by Enterobacteriaceae (Pereira et al., 2004; World Health Organization [WHO], 2017). Failure of treatment with these antibiotics has increasingly been reported due to the emergence of extended spectrum beta-lactamases (ESBLs) during the last two decades (Pitout and Laupland, 2008). Several studies have suggested that children are more likely to be exposed to antibiotics both directly (Alexander et al., 2011; Saari et al., 2015) and indirectly, through exposure to antibiotics taken by their mothers (Verani et al., 2010; Macones et al., 2012; Ledger and Blaser, 2013; de Tejada, 2014). This direct and/or indirect consumption of antibiotics might thus affect infants’ intestinal microflora, including *Escherichia coli*, which is one of the first bacterial species that colonizes the infant gut (Hewitt and Rigby, 1976).

Antimicrobial resistance in commensal bacteria is worrisome due to its ability to spread to pathogens (Munk et al., 2018). Recent studies have showed that school children and children up to 2 years of age were colonized by *E. coli* resistant to broad spectrum antibiotics and ciprofloxacin, respectively (Gurnee et al., 2015; Ferjani et al., 2017). However, there is no information on the carriage rate and abundance of this antibiotic-resistant *E. coli* in relation to the total number of *E. coli* present in the gut. In addition, there is no data available on the carriage rate of MDR *E. coli*, including ESBL-producing *E. coli*, among infants under 1 year old. Therefore, this study evaluated the prevalence and rate of colonization of this organism during the early life of infants. We determined the prevalence, abundance, and carriage rate of 3GC-resistant (3GCr) *E. coli*, including pathogenic *E. coli*, by analyzing culturable *E. coli* in infant’s stool samples.

## Materials and Methods

### Ethics Statement

The research and the ethical review committees of icddr,b approved and monitored the progress of the study. Informed written consent was obtained from mothers of all infants either by signature or, for those who were not literate, by thumbprint after verbal communication. Samples were identified with codes to preserve anonymity. A witness also signed each informed-consent form. All authors vouch for the completeness and accuracy of the data and analyses presented.

### Study Design, Site, and Enrollment of Participants

We conducted a cross-sectional study of children <1 year of age living in five rural villages of Matlab and Hajiganj, sub-districts of Chandpur district of Bangladesh, between March and October 2017. Hajigonj has a total area of 189 km^2^ with 327,367 people living in 64,257 households at 11 unions, whereas Matlab Uttar has a total area of 279 km^2^ with 382,195 people in 62,418 households at 15 unions (DGHS Health Bulletin, 2014). According to the The World Bank (2016) the crude birth rate for Bangladesh is 18.95 per 1000 people as of 2016, so an approximation for the number of infants in Hajigonj and Matlab Uttar is 6,200 and 7,250, respectively, or less than 13,500 total infants (2016). For study sites we included one union from each sub-district. A total of 100 households (50 from each union) containing one infant (age ≤1 year) in each household were enrolled in the study after obtaining written informed consent from the mothers of enrolled infants. A pre-tested survey questionnaire was used to collect information on age, sex, mode of delivery, maternal and child antibiotic consumption, disease morbidity, and feeding practices.

### Sample Collection

A total of 100 stool samples were collected from 100 infants located in the selected households using sterile stool containers provided earlier to all the mothers on the date of the interview. Assuming an infant population in Hajigong and Matlab Uttar of 13,500, the sample size of 100 stool samples would imply a margin of error of approximately 10% with 95% confidence interval for prevalence rates of 3GCr *E. coli* (Dhand and Khatkar, 2014). The field staff collected the samples on the following day and transported it to icddr,b laboratory (Dhaka, Bangladesh) on ice for microbiological analyses within 4 h.

### Enumeration and Isolation of *E. coli*

Both total and 3GCr *E. coli* were enumerated using the drop plate method as described previously (Herigstad et al., 2001). In brief, MacConkey agar plates (Becton Dickson, MD) with and without fixed concentrations of cefotaxime (1.0 μg/mL) were used to enumerate 3GCr *E. coli* and total *E. coli*, respectively. A total of four 10-fold serial dilutions (10^−1^ to 10^−4^) of each stool sample were made and 50 μl from each dilution was inoculated onto MacConkey agar plates with and without antibiotic added to the culture media. All plates were incubated at 37°C for 18 h and the number of colony forming units (CFUs) per gram wet weight of stool sample were counted from the dilution of readable range. Proportion of 3GC-sensitive (3GCs) *E. coli* CFUs per gram feces (CFU/g) count was calculated by subtracting 3GCr CFU/g count from corresponding total *E. coli* CFU/g. Further, proportion of 3GCr *E. coli* count was measured in respect to the total *E. coli* count obtained on MacConkey agar plate. At least three colonies from each sample were confirmed as *E. coli* by API-20E (bioMerieux, France) and stored at −80°C for further characterization.

### Antibiotics Susceptibility Test

Antibiotic susceptibility of *E. coli* (one isolate per sample) was determined by standard disk diffusion technique following the Clinical and Laboratory Standards Institute (CLSI) guidelines (Patel, 2017). The antibiotics used in this study were ampicillin (10 μg), gentamycin (10 μg), tetracycline (30 μg), meropenem (10 μg), imipenem (10 μg), ceftriaxone (30 μg), cefotaxime (30 μg), ceftazidime (30 μg), cefepime (30 μg), colistin (10 μg), ciprofloxacin (5 μg), nalidixic acid (30 μg), azithromycin (15 μg), trimethoprim/sulfamethoxazole (25 μg), nitrofurantoin (30 μg), and chloramphenicol (30 μg) (Oxoid, United Kingdom). The zone of inhibition was measured and the isolates were classified as resistant, intermediate, or sensitive according to the interpretation guideline provided by the CLSI (Patel, 2017). An isolate was considered MDR if resistant to three or more classes of antibiotics.

### Test for Extended Spectrum Beta-Lactamase (ESBL)

Extended spectrum beta-lactamase was tested by combination disk test (CDT) as described by CLSI (Patel, 2017). Disks containing a 3GC, including cefotaxime, CTX (30 μg) or ceftazidime, CAZ (30 μg) alone and in combination with clavulanic acid (CLA, 10 μg) were applied (Oxoid, United Kingdom). The zone of inhibition around the CTX or CAZ disk combined with CLA was compared with the inhibition zone around CTX or CAZ disks alone. The test was considered positive for ESBL-production if the inhibition zone diameter was ≥5 mm larger with CLA than without (Patel, 2017).

### PCR for ESBL Genes and *E. coli* Pathotypes

All 3GCr *E. coli* were tested for ESBL genes considered priorities due to their clinical relevance, specifically: *bla*_CTX–M–group–1_, *bla_SHV_*, *bla*_TEM_, and *bla*_OXA–1_. These genes were tested by PCR using primer sequences and PCR conditions as described previously (Islam et al., 2017). The pathotypes of *E. coli* (EPEC, ETEC, EAEC, EIEC, and STEC) were identified by multiplex PCR of different pathogenic genes according to the procedure described earlier (Talukdar et al., 2013).

### Statistical Data Analysis

Data were entered in SPSS 20.0 (IBM Inc., Chicago, IL, United States). Data cleaning, statistical analysis and graphical presentation were done in Stata 13.0 (College Station, TX, United States) and R-3.4.2 (R Core Team, 2014). *E. coli* concentrations were log_10_ transformed in order to assess the association between demographic variables with 3GCr *E. coli* carriage using chi-square test and non-parametric Mann–Whitney *U*-test. Population counts of the susceptible and resistant isolates from the same infant were compared using Wilcoxon Rank-Sum test on the paired data. Statistical significance was determined using alpha = 0.05 for all tests except for the Wilcoxon Rank-Sum test which stratified analyses by age (1–3, 4–6, 7–9, and 10–12 months). For this, the conservative Bonferroni correction was applied to adjust alpha to 0.0125 (0.5/4) to correct for multiple comparisons.

## Results

### Carriage of 3GCr *E. coli* in Infant’s Fecal Sample

Of the 100 stool samples from infants, 82% were positive for 3GCr *E. coli*. Mean count ± standard deviation for total *E. coli* was 6.86 ± 1.56 log_10_ *E. coli* CFU/g of stool while the mean count of 3GCr *E. coli* was 6.21 ± 1.32 log_10_ CFU/g. On average, 3GCr *E. coli* encompasses approximately one third of (33%) of the total *E. coli* present/g wet weight of stool.

### Antibiotic Susceptibility of 3GCr *E. coli*

**FIGURE 1:**
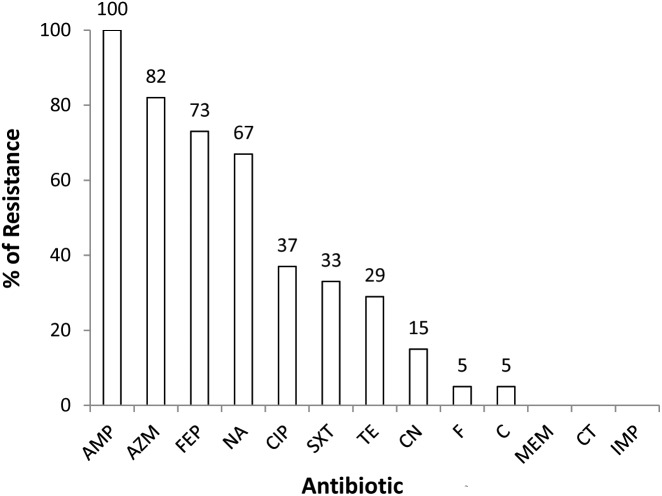
Prevalence of 3GCr *E. coli* resistant to different classes of antibiotics in infants aged from 1 to 12 months (*n* = 82). AMP, ampicillin; AZM, azithromycin; FEP, cefepime; NA, nalidixic acid; CIP, ciprofloxacin; SXT, sulfamethoxazole; TE, tetracycline; CN, gentamycin; F, nitrofurantoin; C, chloramphenicol; MEM, meropenem; CT, colistin; IMP, imipenem; 3GCr, third generation cephalosporins resistant.

All the 3GCr (*n* = 82) *E. coli* isolates were tested for susceptibility against a panel of 13 antibiotics. Resistance to multiple antibiotics other than 3GCr was very common, with 77% (*n* = 63) of isolates classified as MDR. None of the isolates were resistant to colistin or carbapenem (Figure 1).

### Prevalence of ESBLs Among 3GCr *E. coli* Isolates

CDT of all 3GCr *E. coli* (*n* = 82) isolates revealed that more than 90% (*n* = 74) were ESBL-producing. Among ESBL-producing isolates, 96% (*n* = 71) were positive for *bla*_CTX–M–group–1_, 41% (*n* = 30) for *bla*_TEM_, and 11% (*n* = 8) for *bla*_OXA–1_. Of the 8 CDT negative isolates, 4 were positive for *bla*_CTX–M–group–1_ indicating that these isolates might have co-produced ESBL and AmpC enzymes. Given the high rates of positivity for detection of *bla*_CTX–M–group–1_, further molecular characterization for resistance genes was not conducted; carriage of multiple mechanisms of resistance is possible but was not explored.

### Prevalence of *E. coli* Pathotypes Among 3GCr *E. coli* Isolates

Analysis of virulence genes among 3GCr isolates revealed that 23% (*n* = 19) of the isolates were positive for genes specific for EAEC (*aat*A, *aai*C) and 3.6% (*n* = 3) of the isolates were positive for genes specific for EPEC (*bfp*, *eae*). No other virulence genes were detected (*lt*, *st*, *ipa*H, and *ial*).

### Determinants of 3GCr *E. coli* Carriage Among Infants

Statistical analysis of results did not reveal any significant association between the presence of 3GCr *E. coli* in infant stool and characteristics of the infant, including gender, religion, age, mode of delivery, feeding practice of child, diarrheal history, and maternal and child antibiotic consumption (Table 1). Moreover, association between the rate of colonization of 3GCr *E. coli* in the child’s gut and demographic characteristics of infant (age, *p* = 0.5; delivery mode, *p* = 0.8; infant and mother antibiotic consumption, *p* = 0.2, *p* = 0.4; and infant diarrhea, *p* = 0.2) were not statistically significant in non-parametric Mann–Whitney *U*-test (Table 2). Therefore, the presence or abundance of 3GCr *E. coli* in infant stools in this study could not be explained by some of the most common risk factors, including prior exposures to antibiotics.

**Table 1:** Demographic characteristics of infants with and without fecal carriage of third generation cephalosporins resistant (3GCr) *E. coli*.

Characteristics	3GCr *E. coli*		*p*-Value
	Positive, *n* = 82 (%)	Negative, *n* = 8 (%)	
Sex (Male)	44 (54)	3 (38)	0.472
Religion (Muslim/Hindu)	72 (88)	5 (63)	0.087
Age ≤6 months	33 (40)	5 (63)	0.275
Mode of delivery (CS)^∗^	27 (32)	3 (38)	1.000
Mode of delivery (NVD)^∗^	27 (32)	3 (38)	1.000
Exclusively breast feeding	1 (1)	0	1.000
Exclusively formula feeding	1 (1)	0	1.000
Complementary feeding	80 (98)	7 (88)	1.000
Diarrhea (Yes)	9 (11)	0	0.593
Antibiotic consumption (6 months before)	53 (65)	3 (38)	0.149
Other complication (Cold)	47 (57)	2 (25)	0.135
Maternal antibiotic consumption	12 (15)	1 (13)	1.000

*^∗^NVD, normal vaginal delivery; CS, cesarean section. ^∗^Mode of Delivery (CS and NVD) was obtained for 64 cases, and information from the remaining 36 cases was not available. Number in the parentheses indicates percentage.*

**Table 2:** Association between demographic variables and third generation cephalosporins resistant (3GCr) *E. coli* colonization in infants’ gut.

Factors	Characteristics	Frequency (*n*)	3GCr *E. coli* mean count (CFU/g wet weight of stool)	*p*-Value
Infant age	Age <= 6	33	3.2 × 10^6^	0.630
	Age > 6	49	2.1 × 10^6^	
Mode of delivery	NVD	27	3.3 × 10^6^	0.665
	CS	27	2.4 × 10^6^	
Child antibiotics	Yes	53	3.2 × 10^6^	0.259
	No	29	1.6 × 10^6^	
Child diarrhea	Yes	9	1.3 × 10^6^	0.632
	No	70	2.2 × 10^6^	
Maternal antibiotics	Yes	12	4.2 × 10^6^	0.758
	No	70	2.3 × 10^6^	

*NVD, normal vaginal delivery; CS, cesarean section.*

### Age-Wise Distribution of Fecal Carriage of 3GCr *E. coli* in Respect to 3GCs *E. coli*

**FIGURE 2:**
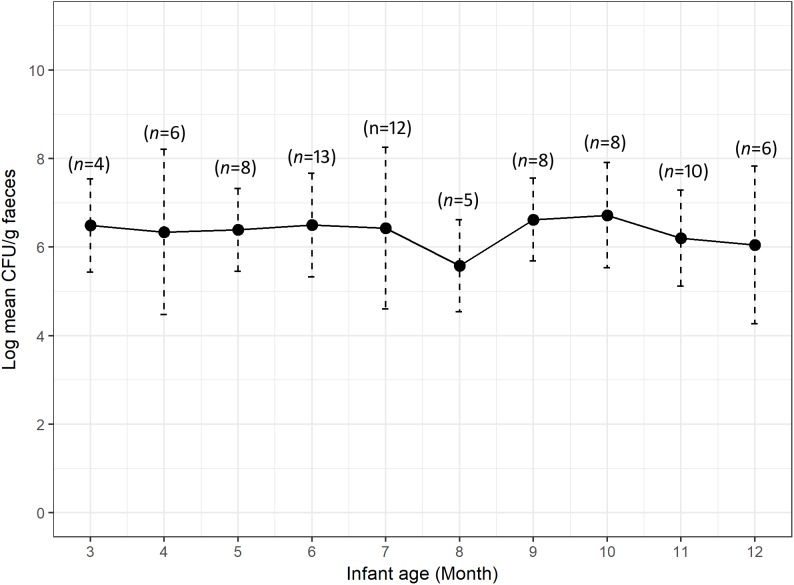
Variations in carriage rates of 3GCr *E. coli* in infants at different ages from 3 to 12 months. The log_10_ mean count of 3GCr CFU from infant at 1 and 2 month of age was excluded because only one infant of each month was obtained for these time periods. Error bars are the standard errors of the results for each age group. 3GCr, third generation cephalosporins resistant.

**FIGURE 3:**
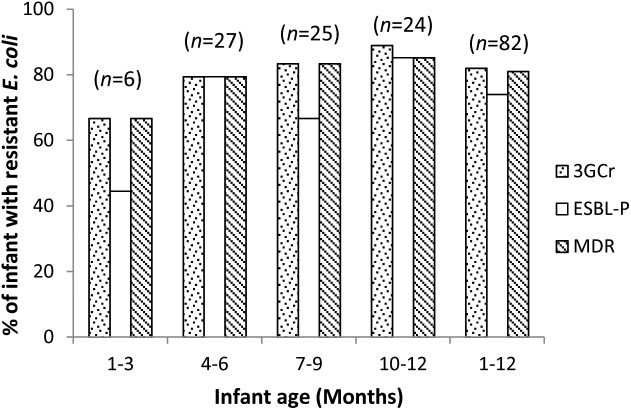
Age wise distribution of prevalence of 3GCr, MDR, and ESBL-P *E. coli* in infant stool samples. 3GCr, third generation cephalosporins resistant; ESBL-P, ESBL-producing; MDR, multidrug-resistant.

We calculated the mean count of 3GCr *E. coli* among infants of the same age groups and plotted the distribution with 1 month intervals in order to determine if the CFU count of resistant microflora changes with infant age. Analysis of mean count of 3GCr up to 12 months showed that log_10_ CFU mean count of 3GCr even at 3 months of age was high (6.43 log_10_) and consistent in subsequent months (Figure 2), indicating early appearance of 3GCr *E. coli* in infants. However, the percentage of infants infected with resistant *E. coli* population (3GCr and MDR) was progressively increased as infant age grown by months (Figure 3).

**FIGURE 4:**
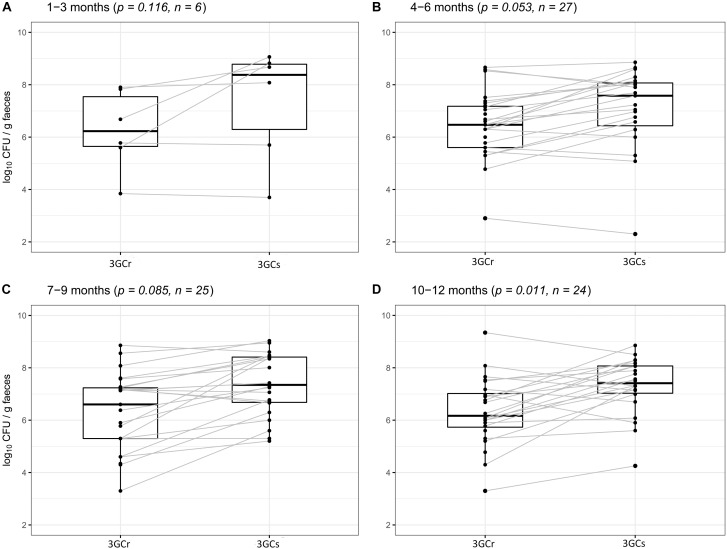
Comparative analysis of carriage rates (in CFU/g wet weight of stool) of 3GCr and 3GCs *E. coli* within similar age group of infants. **(A)** 1–3 months of age, **(B)** 4–6 months of age, **(C)** 7–9 months of age, and **(D)** 10–12 months of age. 3GCr, third generation cephalosporins resistant; 3GCs, third generation cephalosporins sensitive; CFU, colony forming unit.

We compared the differences between 3GCr and 3GCs *E. coli* counts among infants of the same age groups at 1–3, 4–6, 7–9, and 10–12 months of age to examine whether 3GCr *E. coli* co-exist with the 3GCs favorably without selective pressure of antibiotics. There was no significant difference in the population of 3GCr and 3GCs *E. coli* using Wilcoxon Rank-Sum test at any age group except for the oldest one, 10–12 months (*p* = 0.011) (Figure 4), suggesting that 3GCr *E. coli* can stably persist like 3GCs *E. coli* from early months of life. Notably, statistical significance in the difference in 3GCr and 3GCs *E. coli* amongst infants 10–12 months old is borderline significant compared to the Bonferroni adjusted significance level of alpha = 0.0125 for the age-stratified Wilcoxon Rank-Sum test.

## Discussion

We found an extremely high prevalence of both 3GCr *E. coli* (82%) and ESBL-producing *E. coli* (74%) in stool samples of healthy infants living in rural areas of Bangladesh. Despite the relatively small sample size (*n* = 100) chosen based on logistic constraints, the high prevalence rates are likely representative of Bangladeshi infants under 1 year old in the study area within a margin of error of 10% (with 95% confidence interval). Even with the associated uncertainty, this is the highest prevalence of ESBL-producing *E. coli* in healthy human guts observed to date. For example, in a study in Tunisia, the prevalence of MDR *E. coli* was 6.6% in children aged between 6 and 12 years (Ferjani et al., 2017). The rates of 3GCr *E. coli* in healthy children at various ages was reported as 2.9% in Sweden, 2.7% in Portugal, and 10% in Senegal (Guimaraes et al., 2009; Kaarme et al., 2013). The fecal carriage rate of ESBL-producing Enterobacteriaceae in healthy children (0–59 months) was much higher (59%) in central Africa (Farra et al., 2016). None of these studies have reported the data on culturable 3GCr *E. coli* as a proportion of total culturable *E. coli* in stool samples. In our study we found that around one third of the total *E. coli* colonies obtained in culture were 3GCr, which is alarming.

The difference between 3GCr and 3GCs *E. coli* gives an indication of fitness costs for the maintenance of resistance within the gut microbial community. The proportion of 3GCr amongst the total *E. coli* (sum of 3GCr and 3GCs *E. coli*) was not significant in infants at different age groups, indicating that the competitiveness of resistant bacteria with normal residential flora within the gut is not influenced by age. There may be a low fitness cost associated with persistence and dissemination of resistance. Indeed, Cottell et al. (2012) suggest a low fitness cost associated with plasmid (pCT) carrying the resistance gene *bla*_CTX–M–14_ for *E. coli*, as the *E. coli* were able to persist and disseminate readily even in the absence of selective pressure from antibiotics (Cottell et al., 2012). Our findings also displayed congruence with previous reports demonstrating that tetracycline- and ampicillin-resistant isolates persist continuously without any selective pressure of antibiotics in the gut of different age groups of children (Karami et al., 2006, 2008).

The implication of this high load of 3GCr *E. coli* is substantial in the context of child health safety. Antibiotic resistant *E. coli* and other common commensals of the gut including *Klebsiella* spp. and *Acinetobacter* spp. are amongst the leading causes of community-acquired serious infection in Southeast Asia. In a cross sectional study at five sites across Southeast Asia, Saha et al. (2018) found that only 17% of possible serious bacterial infections (pSBI) identified in young children were resistant to first line antibiotics. Resistant infections, as compared to susceptible infections, are linked with worse outcomes. For example, in Tanzania, children with septicemia caused by bacteria producing extended-spectrum beta-lactamases were almost twice as likely to die compared to non-ESBL infections (71% mortality rate vs. 39%) (Blomberg et al., 2007). In the present study, the observed high carriage rate and high relative proportion of culturable 3GCr *E. coli* may harbinger higher rates of 3GCr *E. coli* as a proportion of pSBI infections. Further research linking fecal carriage of resistant bacteria to risks of resistant infections is warranted.

Apart from increasing the risk of resistant infections, high carriage of 3GCr Enterobacteriaceae in the gut results in shedding through infant stool and thus contributes to an elevated risk of exposure to nearby people and animals. There is a common belief among illiterate or less literate mothers in the community that infant stool is not a health hazard or harmful, especially in comparison to adult stool (Yeager et al., 1999; Gil et al., 2004). According to a national survey during the period from 2012 to 2013 in Bangladesh, feces of about 61% children of age 0–2 years were disposed unsafely where the percentage was much higher in rural areas (67%) compared to urban areas (40%) (Bangladesh Bureau of Statistics [BBS] and United Nations Children’s Fund [UNICEF], 2014). Thus household members including mothers or caregivers are exposed to fecal MDR bacteria through unsafe disposal of infant feces. Similarly, improper disposal of infant’s stool in front yards or nearby ditches may contribute to transmission of these resistant bacteria to domestic and wild birds and/or other animals (Hasan et al., 2012).

The 3GCr *E. coli* isolates in this study were predominantly resistant to azithromycin and ciprofloxacin, among other antibiotics (Figure 1). Azithromycin is the first line of choice for treatment of shigellosis in children (Centers for Disease Control and Prevention [CDC], 2006) and a second line of choice for treatment of shigellosis in adults (World Health Organization [WHO], 2005). Although no infants were reported to be suffering from shigellosis during the study period, shigellosis has been identified as a major contributor to moderate-to-severe diarrheal disease in neighboring Mirzapur (Kotloff et al., 2013). Management of this infection might be complicated due to the high prevalence of azithromycin resistance in the study community. Among other antibiotics, ciprofloxacin resistance was found among 37% of 3GCr isolates, which is even higher than a previous report that showed resistance in 19% of *E. coli* obtained from healthy children (Gurnee et al., 2015). Interestingly, only 29% of *E. coli* isolates in this study were resistant to tetracycline, a first generation antibiotic which is less commonly used for the treatment of *E. coli* infections in the community (Calva et al., 1996; Domínguez et al., 2002). Tetracycline is not prescribed in children due to its effect on the growth of bones and teeth (Sánchez et al., 2004). It suggests that by reducing the use of antibiotics in humans and animals, it is possible to reduce the burden of resistant microorganisms and this can eventually restore the efficacy of the existing antibiotics even in a setting like Bangladesh where overuse of antibiotics and burden of AMR, both are way too high.

Our study demonstrated that infant’s guts serve as a reservoir of *E. coli* resistant to multiple antibiotics including 3GCr and fluoroquinolones, which are critical for the treatment of many infectious diseases in humans (World Health Organization [WHO], 2017). High rates of ESBL-producers among 3GCr isolates in the present study is alarming because patients with community acquired infections as well as their household members carrying ESBL-producing Enterobacteriaceae may spread resistance to other people in their community (Valverde et al., 2008). This can be explained by the overall high prevalence of ESBL *E. coli* infections in the community. A recent study in Bangladesh has reported that 34% of all clinical isolates of *E. coli* from patients with extra-intestinal infections were ESBL-producing (Khan et al., 2018).

The probable cause of colonization with ESBL-producing Enterobacteriaceae among pre-school children in Laos was reported due to misuse of antibiotics (Stoesser et al., 2015). In our study, we did not observe this: there was no significant association between colonization and reported previous use of antibiotic treatment among infants. Previous studies have suggested that acquisition of antimicrobial resistant bacteria or antimicrobial resistant genes in the infant gut might occur during and/or after the delivery (Zhang et al., 2011). Specifically, mothers’ flora during normal vaginal delivery or environmental flora during caesarian (C-section) delivery colonize the infant gut (Zhang et al., 2011). Therefore, AMR bacteria from the mother or hospital environment may contribute to infant carriage. For example, a study carried out in Tunisia showed that 20% of patients acquired 3GCr *E. coli* in their gut due to nosocomial infection (Maamar et al., 2016). In our study, we did not find any significant differences in the level of colonization with 3GCr *E. coli* between infants with normal vaginal delivery and infants delivered through C-section (Tables 1, 2). In Bangladesh, a recent study showed that delivery by C-section increased from 3.5 to 23% between 2004 and 2014 (Khan et al., 2017) and it is a common practice that prophylactic antibiotics are used before and after the surgery. During post-operative care mothers start to breastfeed the newborn while still on antibiotic treatment. This leads to transfer of antibiotics in its active form to infants and thus their gut microbiota may shift to survive in an antibiotic selective environment (Mathew, 2004). Further in Dhaka, Bangladesh, a significant proportion of newborns (98%) receive antibiotics (sulfonamides, fluoroquinolones, metronidazole, penicillins, etc) before 6 months of age (Rogawski et al., 2017), which renders the selective environment for antibiotic-resistant bacteria. Even in a very low concentration of antibiotics, fitness cost for microorganisms to become resistant is lower than becoming antibiotic susceptible (Sandegren, 2014). Surprisingly, in our study neither history of antibiotic use or previous record of hospitalization was associated with 3GCr colonization nor were gender, religion and feeding practices. The lack of association may be due to the high rate of colonization combined with relatively small sample size (*n* = 100). The low proportion of infants without 3GCr limits statistical analyses. Therefore, further studies, particularly focused on larger sample sizes, are needed to identify the causes of the high rate of antibiotic resistance carriage among infants under 1 year old in this setting.

## Conclusion

The high rate of intestinal carriage with MDR microorganisms among infants in rural Bangladesh is a serious concern that can jeopardize the management of infectious diseases. In addition, shedding of high number of MDR microorganisms through infant feces increases the risk of widespread transmission of these microorganisms in the community and environment. This study raises important questions about how the acquisition of resistant microorganisms takes place in infants’ guts within the first 3 months of life, what are the major drivers of acquisition, and what are the implications on infant health and well-being. Future studies should explore the source of acquisition of resistance in infants, to understand whether such resistance is primarily acquired from the environment, vertically from the child’s mother, or through selective pressure from pediatric antibiotic use.

## Author Contributions

MAI and TJ conceived the development. MAI, TJ, MK, KL, TN-D, and MM designed the study and developed the protocol. MA, SR, and MBA contributed to the experiments, collection, and assembly of the data. MRI, MBA, and TJ contributed to the data entry and statistical analysis. MAI and MBA performed the first draft of the manuscript. TJ, MK, KL, TN-D, MM, MA, and MAI revised the manuscript and prepared the final draft of the manuscript.

## Conflict of Interest Statement

The authors declare that the research was conducted in the absence of any commercial or financial relationships that could be construed as a potential conflict of interest.
